# Energy spectrum and quantum phase transition of the coupled single spin and an infinitely coordinated Ising chain

**DOI:** 10.3762/bjnano.16.117

**Published:** 2025-09-24

**Authors:** Seidali Seidov, Natalia Pugach, Anatolie Sidorenko

**Affiliations:** 1 HSE University, Moscow, Russia; 2 NUST MISIS, Moscow, Russia; 3 Technical University of Moldova, Institute of Electronic Engineering and Nanotechnologies, Republic of Moldova; 4 Moscow Institute of Physics and Technology, Dolgoprudny, Russia

**Keywords:** Ising chain, Lipkin–Meshkov–Glick model, quantum phase transitions

## Abstract

In this work we consider a spin model composed of a single spin and connected to an infinitely coordinated Ising chain. Theoretical models of this type arise from various fields of theoretical physics, such as theory of open systems, quantum control, and quantum computations. In the thermodynamic limit of an infinite chain, we map the chain Hamiltonian to the Hamiltonian of the Lipkin–Meshkov–Glik model, and the system as a whole is described by a generalized Rabi Hamiltonian. Next, the effective Hamiltonian is obtained using the Foulton–Gouterman transformation. In the thermodynamic limit we obtain the spectrum of the whole system and study the properties of the ground-state quantum phase transition.

## Introduction

In the present manuscript, we consider a single spin connected to an infinitely coordinated Ising chain. From a purely theoretical point of view, this model arises when studying the physics of open systems [1–2]. In this case, the chain is modelling the external environment to which the single spin is connected. In such models, it is convenient to study not only Markovian dynamics of the single spin, but also non-Markovian dynamics going beyond the limitations of the Lindblad master equation [3–6]. The approach is to find the dynamics of the whole system (i.e., the chain and the single spin) and then trace out the chain degrees of freedom, ending up with the master equation for the single-spin density matrix. One might choose to make or not to make the Markov approximation, obtaining different types of master equations. Given that the exact solution is known, different master equation solutions can be compared against it. This allows to study the limits of applicability of the Markovian approximation, and also the correct way of introducing the Lindblad dissipation operators. These problems remain important in the general field of open quantum systems, extending beyond spin models [7–9].

One of the practical applications is modelling of certain quantum computing layouts, if one considers spins as qubits. In particular, we have previously proposed a method for implementing a CCZ (control–control–Z) quantum gate on a system composed of three logical qubits, which are connected to another coupler qubit [10]. This approach allows to increase the fidelity of the operation, and it has technical benefits such as simplicity of calibration and suppression of the unwanted longitudinal ZZ interaction. One of the important quantities is the shift of the coupler qubits energy levels depending on the state of the logical qubits. In the present manuscript, we find the energy levels of such system in the limit of infinitely many logical qubits, and find the energy spectrum of the coupler qubit depending on the state of the logical qubits ensemble.

We start our theoretical analysis by mapping the Ising chain Hamiltonian to a Lipkin– Meshkov–Glik (LMG) Hamiltonian [11–13]. The Hamiltonian of the whole system then becomes akin to the Hamiltonian of the generalized Rabi model, but with the bosonic field replaced by the collective spin of the LMG model. Next, it is diagonalized in the spin space using the Fulton–Gouterman transformation and we obtain an effective Hamiltonian. In the limit of infinite Ising chain, or equivalently of the infinite total spin, the LMG Hamiltonian can be solved exactly. We exploit this fact and analytically obtain the energy spectrum of the whole system. Based on this result, we study the structure of the extrema of the ground state energy and the consequent properties of different phases of the system.

## Model

We consider a single spin coupled to a fully connected Ising chain with the Hamiltonian


[1]

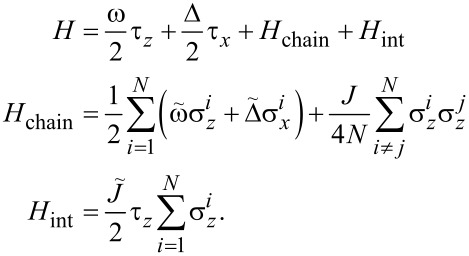



Here, τ*_x_*_,_*_z_* are the Pauli matrices describing the single spin, and 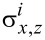 are the Pauli matrices describing spins in the Ising chain. This model arises when one studies spin-bath theoretical models in studies of quantum control and design of qubit layouts in quantum computation. The coupling between the spins in the chain is rescaled by 1/*N* factor in order to obtain a finite energy per spin ⟨*H*⟩/*N* in the thermodynamic limit.

Let us first consider the Hamiltonian *H*_chain_ + *H*_int_. By introducing collective spin operators


[2]

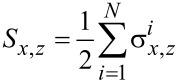



the Hamiltonian is brought in the following form [14–15]:


[3]

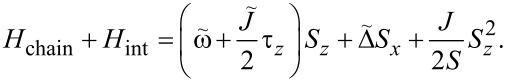



Here, *S* = *N*/2 is the total spin of the chain. This is a well-known Lipkin–Meshkov–Glick (LMG) Hamiltonian which we will further denote as *H*_LMG_ = *H*_chain_ + *H*_int_. The total Hamiltonian now can be written as a 2 × 2 block matrix in the single-spin Hilbert space:


[4]

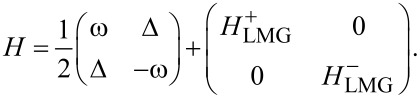



Here 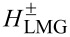 are the Hamiltonians *H*_LMG_ corresponding to eigenvalues ±1 of τ*_z_*. These types of Hamiltonians are the Hamiltonians of the generalized Rabi models. These describe a two-level system connected not to a single bosonic mode, but to a more complicated environment [16–18].

## Diagonalization in the Spin Space

The Hamiltonian in the spin space can be diagonalized using the formula for the determinant of a 2 × 2 block matrix. This is also known as the Fulton–Gouterman transformation [19]. This leads to two effective Hamiltonians in the chain Hilbert space, corresponding to the state of the single spin. These are:


[5]

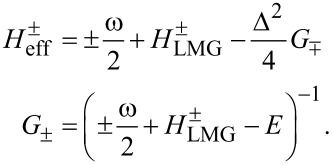



Operators *G*_±_ are the Green functions of the Hamiltonians 
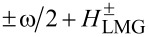
. Both of these Hamiltonians contain full information about the system, so it is sufficient to consider only one of them. We will choose the Hamiltonian 
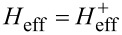
 as the effective Hamiltonian. Given the eigenenergies 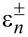 and eigenstates |*n*^±^⟩ of the Hamiltonian 
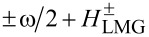
, the effective Hamiltonian can be written as:


[6]

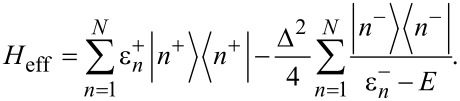



The eigenenergies of the whole system are solutions of the equation λ(*E*) = *E*, where λ(*E*) are the eigenvalues of *H*_eff_. In principle, solutions of this equation are exactly the energy levels of the corresponding physical system. However, given that in practice an analytical solution is impossible in most cases, a usual approach is to substitute some values of energy *E*_0_ on the left hand side and look for corrections. Our approach will be to find some kind of relation between the Hamiltonians 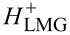 and 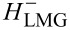, which will allow us to express the eigenstates of one Hamiltonian via the eigenstates of the other. Then, the equation λ(*E*) = *E* will be quadratic with two solutions, corresponding to two states of the single spin.

## Limit of Strong Single-Spin–Chain Coupling

We focus on the limit of large coupling between the single spin and the chain (i.e., large 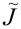). In practice, this can be realized by coupling a single spin to an ensemble of noninteracting spins, such that the ensemble interacts only indirectly through the external spin. In this case, spins in the chain are mostly aligned along the *z*-axis due to the large 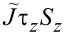 term. Effectively, an interaction with the single spin creates a strong magnetic field parallel to the single spin direction. Thus, the perpendicular component of the “magnetic field” 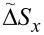 can be considered as a small perturbation. Formally, this means that we can divide the LMG Hamiltonian into the main part


[7]

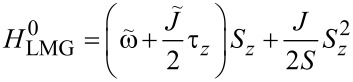



and the perturbation 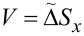. With the standard perturbation theory approach, we find the energy levels of *H*_LMG_ up to second order in 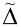:


[8]

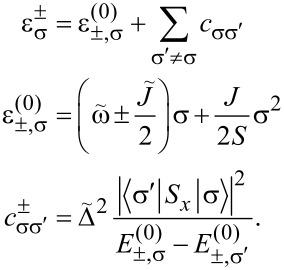



Here, *S_z_*|σ⟩ = σ|σ⟩. Accordingly, the eigenstates are


[9]

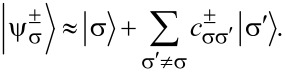



As discussed earlier, we aim to relate 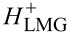 and 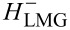. Let us express the projectors on states 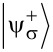 via projectors on 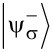. Up to the second order in 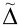
_:_


[10]

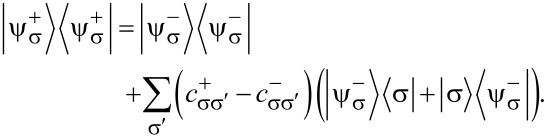



The Hamiltonians 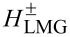 now can be written as:


[11]

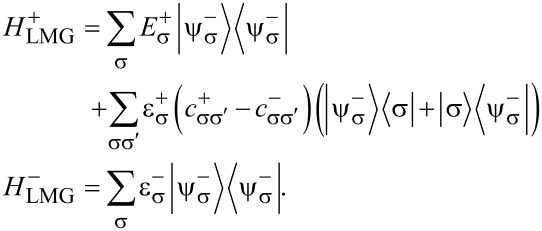



One can see that the leading order 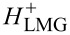 is expressed via projectors on the eigenstates of 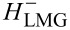. The extra terms, when substituted in the effective Hamiltonian, will lead to higher order corrections and will be insignificant. Indeed, substituting in (Equation 6) we find:


[12]

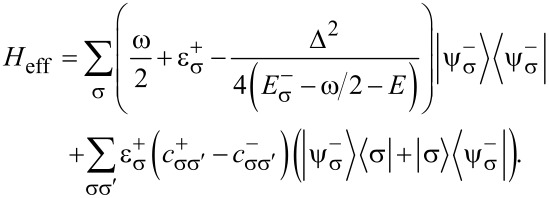



The first term is diagonal in the basis 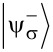, so its contribution to the eigenvalues of the effective Hamiltonian eigenvalues will be of second order in 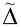 (as it is the order to which we have expanded 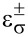). The second term is of second order in 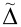 and off-diagonal, so its contribution will be of fourth order in 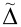. Thus, up to the second order in 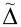, the energy *E* of the whole system is defined by the following equation:


[13]

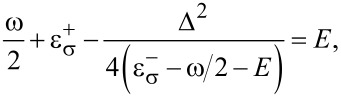



from which follows


[14]

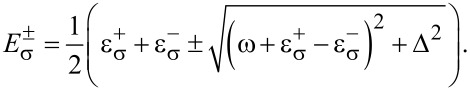



Also, from these calculations follows that the eigenstates are 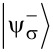. One might wonder why there is no contribution from 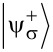, given that our choice between expanding the Hamiltonian (Equation 13) in 
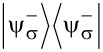
 or 
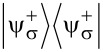
 was arbitrary. In fact, there is indeed no difference between choosing one over the other, because 
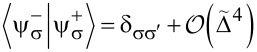
.

We also note that the same spectrum corresponds to the single-spin Hamiltonian


[15]

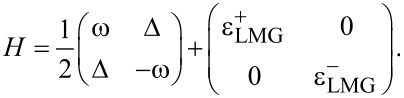



This Hamiltonian can be obtained if one replaces 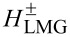 by their eigenvalues 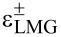 in Equation 4. This is a Born–Oppenheimer approximation in which the chain is considered to be a fast subsystem relative to the single spin. In particular, the energy of the spin chain is a contribution to the potential energy of the single spin.

## Phase Transition in the Thermodynamic Limit

### Phase transition of the bare LMG model

In the thermodynamic classical limit, the spin operators in the LMG model can be replaced by classical expectation values (i.e., *S_z_* = *S*cosθ, *S_x_* = *S*sinθcosφ, *S_y_* = *S*sinθsinφ). The Hamiltonian is then replaced by its classical energy profile, which is defined according to [12] as:


[16]

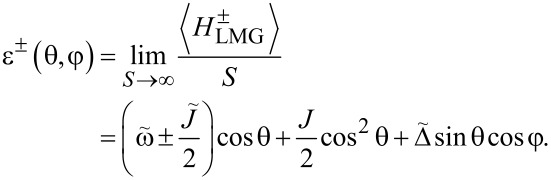



**Figure 1:**
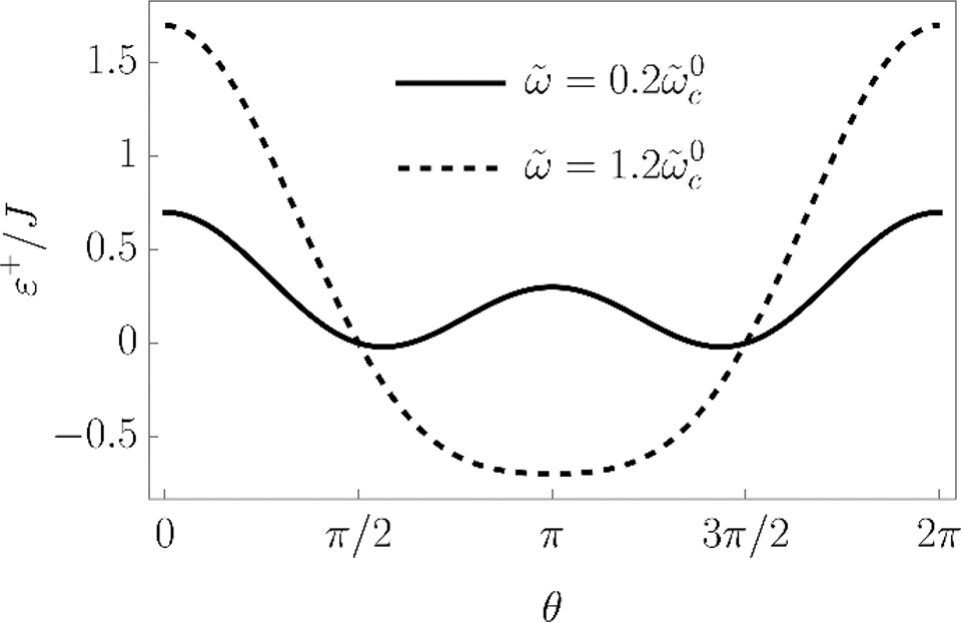
Energy profile (Equation 16) of the LMG model in the thermodynamic limit as a function of θ at φ = 0 and 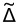 = 0. The solid line corresponds to the broken symmetry phase with two stable minima, and the dashed line to the symmetric phase with a single minimum at θ = π.

The average is taken over a spin coherent state |θ,φ⟩. It is known that the LMG Hamiltonian has two distinct phases in the thermodynamic limit [12,20–22]. The symmetric phase, in which |⟨*S_z_*⟩| = *S*, is realized when the linear part in *S_z_* term in the Hamiltonian dominates over the quadratic one. In our particular case, this means competition between the values of coefficients 
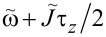
 and *J* in the Hamiltonian (Equation 3). The second broken symmetry phase, in which the energy profile has two minima at 
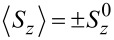
, is realized in the opposite case, when the 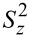 term dominates over the ~*S_z_* term. These minima are degenerate if 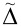 = 0, otherwise one is lower than the other. The plot of the LMG model energy as a function of the angle θ is presented in Figure 1.

We wished to study the phase transition of the bare LMG model (i.e., decoupled from the external spin) and in the next section we will compare the results with the ones for the LMG model coupled to the external spin. First, we have to find the extrema of the LMG model energy 
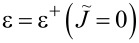
. They are defined by the following equations:


[17]

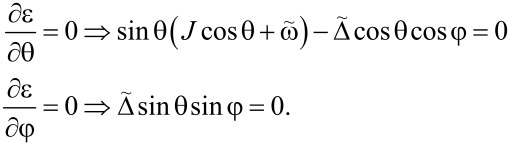



One of the solutions is sinθ = 0 and cosφ = 0; it corresponds to the symmetric phase in which |⟨*S_z_*⟩| = |*S*cosθ| = *S*. The second solution corresponds to sinφ = 0 and


[18]

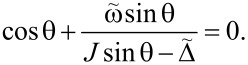



It describes the broken symmetry phase in which |⟨*S_z_*⟩| ≠ *S*.

We found the critical values of the parameters, at which the phase transition occurs, by checking the stability of the symmetric phase. Namely, if it is stable, the sinθ = 0 and cosφ = 0 extremum is a minimum in the θ direction, and the second derivative of ε^+^(θ,φ) with respect to θ is positive. Otherwise, the said extremum is a maximum and the stable phase is the broken symmetry one. When carrying out the calculations, we should choose the θ = π solution of the equation sinθ = 0, since the ~*S_z_* contribution to the energy is positive and the ground state corresponds to *S_z_* = cosπ = −*S*. The θ = 0 solution corresponds to the maximum of the energy profile. Thus, we find:


[19]

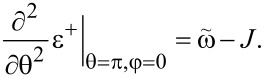



Therefore, the broken symmetry phase exists (i.e., the expression above is negative) for 
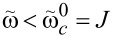
. The projection of the spin on the *z*-axis in the broken symmetry phase is 
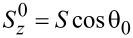
, where θ_0_ is the solution of Equation 18. If 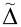 = 0, the solutions are 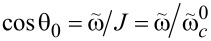. The plots of the energy profile ε^+^(θ,φ) in symmetric and broken symmetry phases are presented in Figure 1.

### Phase transition of the LMG model coupled to a single spin

Next, we study the properties of the phase transition if the chain is coupled to the external single spin. In this case, we have to minimize the ground state energy of the whole system. From Equation 14 we find the spectrum:


[20]

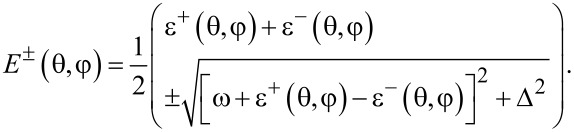



**Figure 2:**
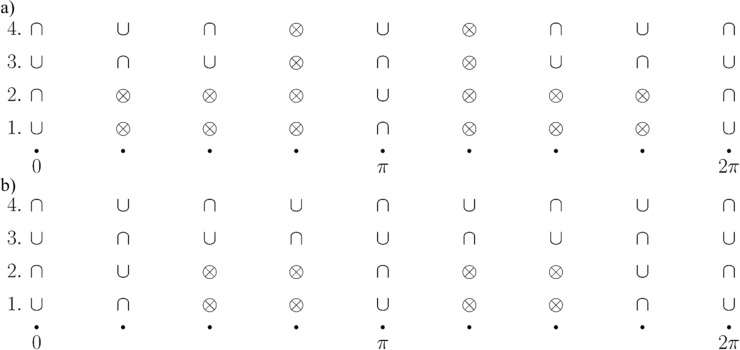
Possible configurations of the extrema of the ground state energy *E*^−^(θ,φ). The symbol ∪ denotes a minimum, ∩ – a maximum, and 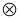 – absence of the extremum. In (a) “different type” configurations are presented and in (b) “same type” configurations are presented.

These functions also have nontrivial minima structure depending on the values of the parameters, see Figure 2. Again, from equations ∂_θ_*E*^−^ = 0 and ∂_φ_*E*^−^ = 0 we find that the extrema of the ground state energy are defined by equations


[21]

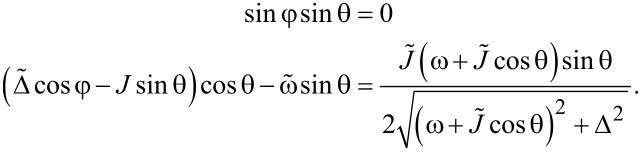



The second equation defines ⟨*S_z_*⟩ in the broken symmetry phase, analogously to Equation 18. In general, it has up to nine real solutions on the interval θ ∈ [0,2π] depending on the values of the parameters. For 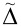 = 0, three of them are θ = 0, π, 2π as it follows from the condition sinθ = 0. Nonzero 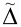 will shift these solutions and corrections due to small 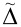
which can be found by expanding the equation at said points. Six other solutions cannot be analytically found; however, we can study the properties of the energy profile exploiting the following facts: 1) due to the 2π-periodicity of E^±^(θ,φ), the extrema at θ = 0, 2π are of the same type. 2) Three of unknown extrema are on the interval θ ∈ (0,π) and the other three are on the interval θ ∈ (π,2π). 3) A maximum should be followed by a minimum and vice versa.

Additional extrema arise due to hybridization between the energy levels ε^±^(θ,φ) of the bare LMG model with the single spin directed up or down, leading to the appearance of avoided crossings and richer extremum structure of the ground state energy. The extrema at θ = 0, π, 2π always exist and could be either minima or maxima and the six other ones might be minima, maxima, or not exist. This allows us to list all possible extrema configurations of the energy profile. We group them into two types: either minima at θ = 0, 2π and θ = π are of different types or the same, see Figure 2a and Figure 2b, respectively.

**Figure 3:**
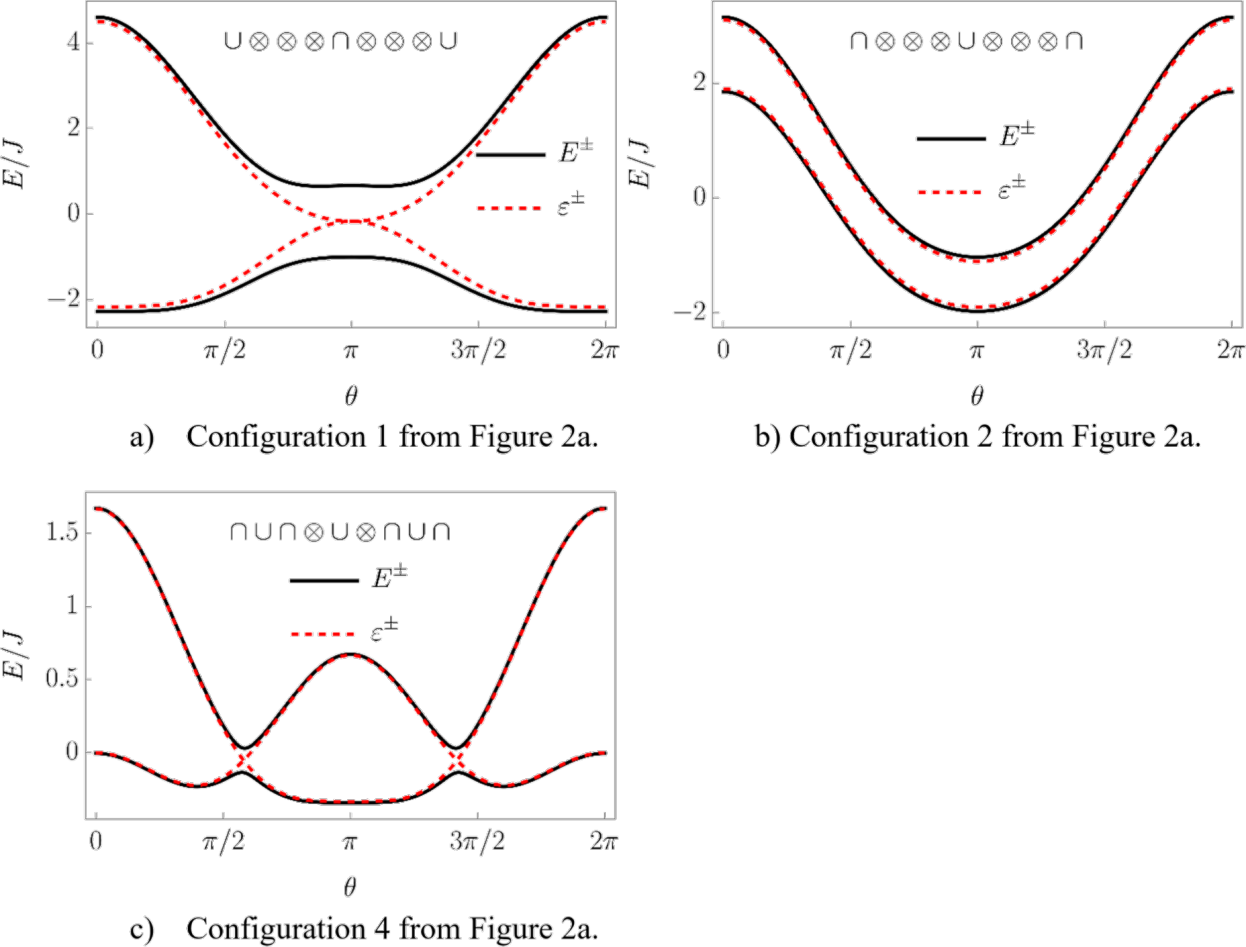
The energy levels of the system corresponding to configurations 1, 2, and 4 from Figure 2a. In plots a) and b) the symmetric phases with spin aligned along positive and negative directions of the *z*-axis, respectively, are stable. In plot c) one can observe a stable symmetric phase and a metastable broken symmetry phase.

Let us first start with “different type” configurations in Figure 2a. Configurations 1 and 2 have no additional extrema and in both of them the symmetric phase is stable. The difference is that in configuration 1 the total spin of the chain is aligned along the positive direction of the *z*-axis, and in configuration 2 – along the negative direction. Remarkably, configuration 1 is unstable for the bare LMG model. Configuration 3 is not realized and in configuration 4 both symmetric and broken symmetry extrema are minima, meaning that one of the phases is stable and the other is metastable. This means that coupling to the external spin can change the type of the phase transition between two phases from the second to the first one. The corresponding plots of the energy levels are presented in Figure 3.

**Figure 4:**
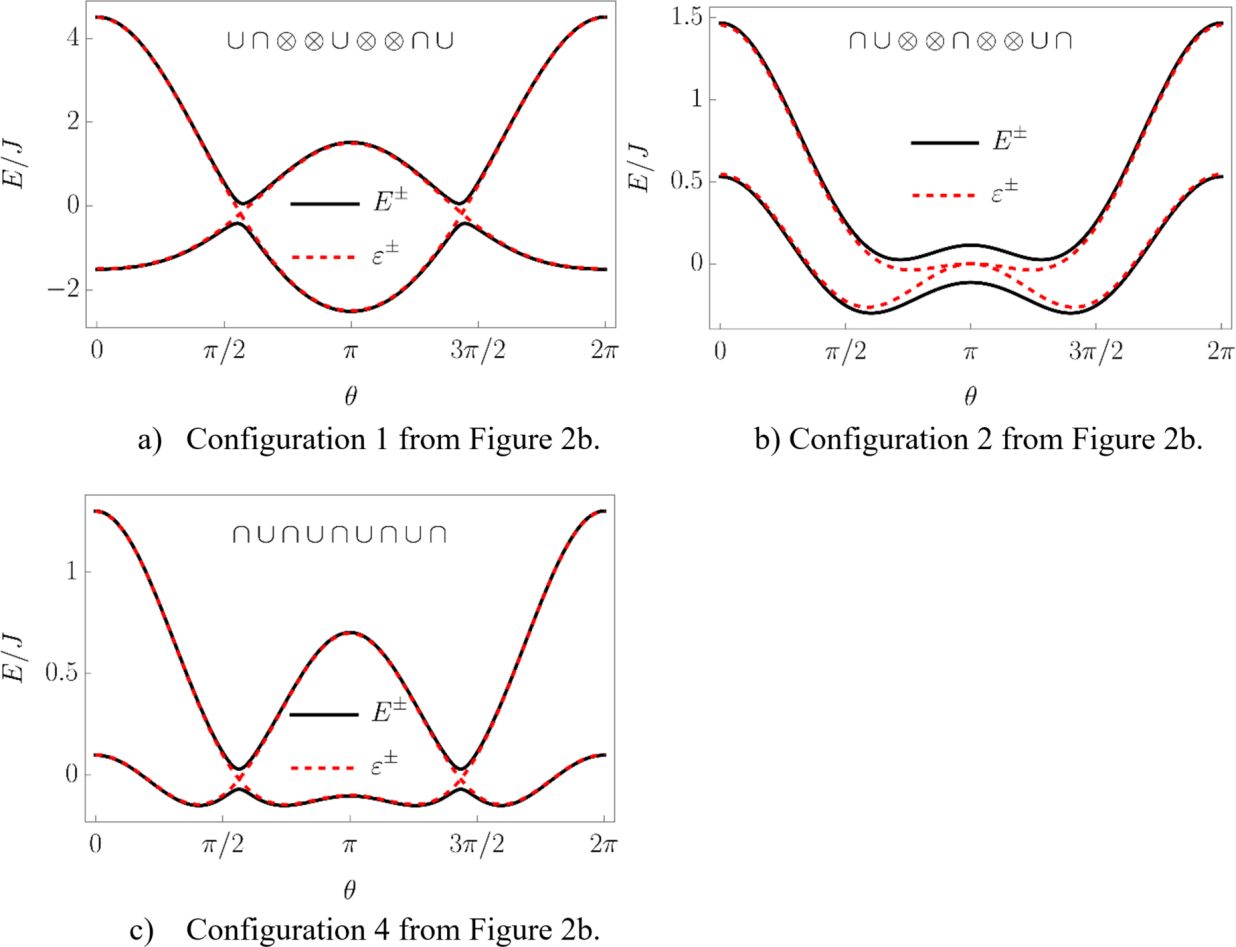
The energy levels of the system corresponding to configurations 1, 2, and 4 from Figure 2b. In plot a) the symmetric phases with spin aligned along negative direction of the *z*-axis is stable and the one with spin aligned along the positive direction is metastable. In plot c) the minima corresponding to the stable broken symmetry phase are split into two.

Now we consider the “same type” configurations in Figure 2b. In configuration 1, two minima correspond to symmetric phases with the total spin aligned along the positive and negative directions of the *z*-axis. One of the phases is stable and the other is metastable, so a first order phase transition between them is possible. In configuration 2, the broken symmetry phase is stable, resembling the case of the bare LMG model. The configuration 3 is again not realized. The most interesting one is the configuration 4 in which the minima, corresponding to the stable broken symmetry phase, split into two. The plots of the energy levels, corresponding to described extrema configurations, can be found in Figure 4.

We also derive the conditions for stability of the points θ = 0, π, 2π. Calculating the second derivatives we find:


[22]

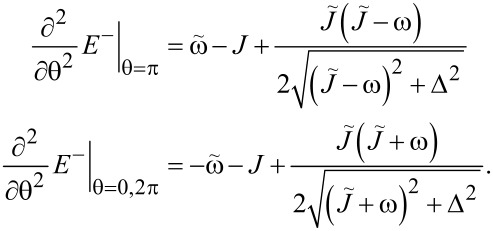



Accordingly, the point θ = π is a minimum if


[23]

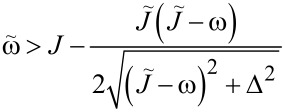



and points θ = 0, 2π are stable if


[24]

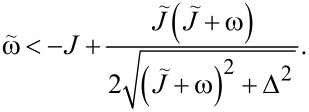



**Figure 5:**
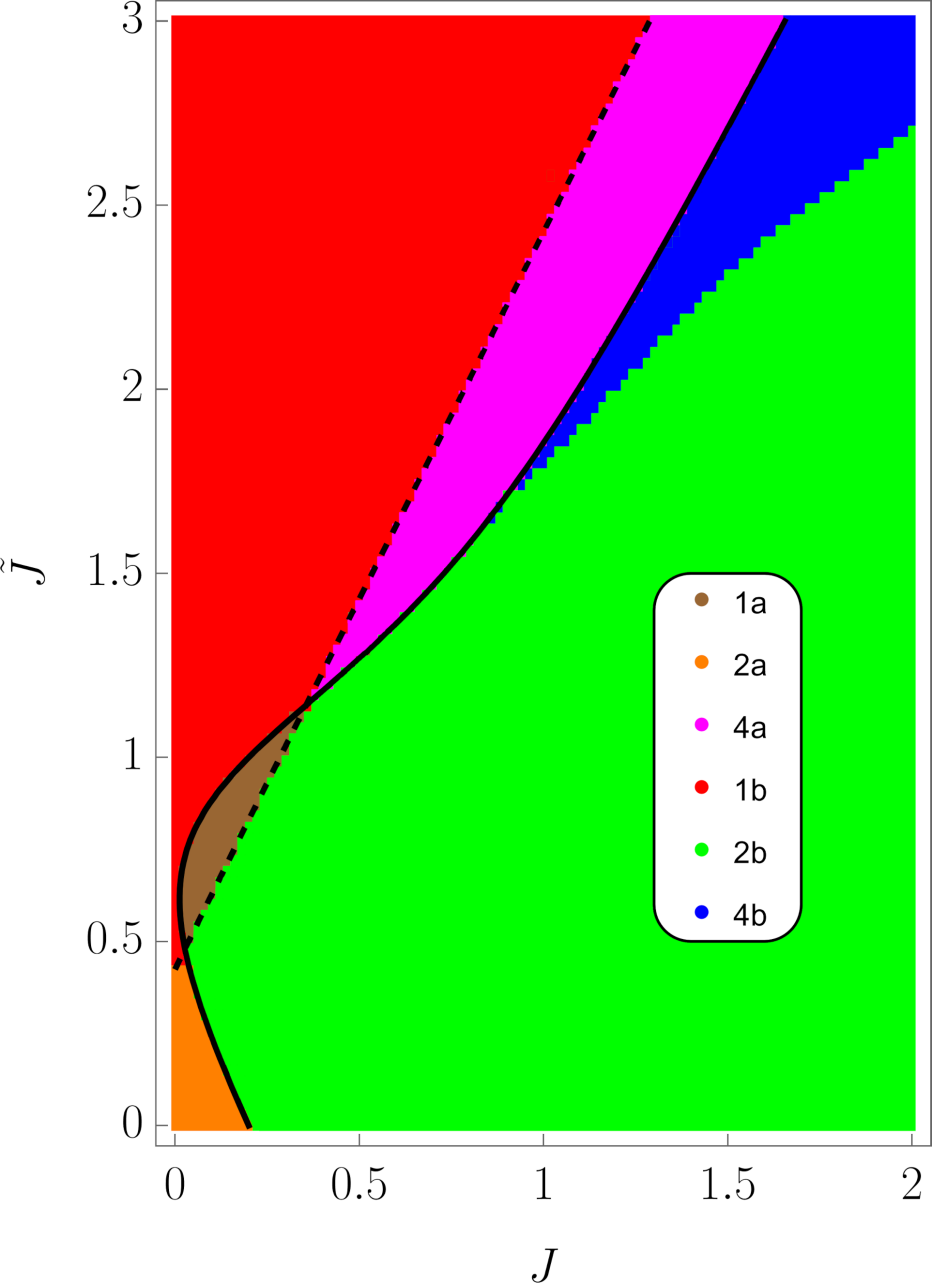
The phase diagram of the considered model on the 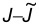 plane. The legend notation coincides with the configurations in Figure 2 (e.g., 1a is the configuration 1 in Figure 2a). The black solid line is the contour given by Equation 23, and the dashed black line is given by Equation 24. The rest of the parameters are chosen as ω = 1, 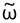 = 0.2, Δ = 0.5, and 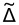 = 0.

Two important observations can be made here. First, the right-hand side of Equation 23 can be negative for large enough values of 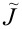 (i.e., single spin–chain coupling) making the symmetric state always stable. Second, at certain range of parameters, both conditions (Equation 23) and Equation 24 might be true or not true simultaneously, which leads to the appearance of the “same type” configurations. More detailed analysis of transitions between different phases requires knowing the conditions of existence of intermediate extrema at θ = 0, π, 2π. This boils down to finding all solutions of Equation 21 and one has to resort to numerical calculations. In Figure 5, the phase diagram, obtained numerically, is presented on the 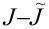 plane for fixed values of the rest of the parameters.

## Results and Discussion

We have theoretically studied the infinitely coordinated Ising chain, coupled to a single external spin. We have written down the effective Hamiltonian in the Ising chain space by diagonalizing the Hamiltonian of the whole system in the space of the external spin. In the thermodynamic limit, when the chain Hamiltonian is exactly solvable, the energy spectrum of the system was found. It is demonstrated that coupling to an external spin drastically changes the properties of the phases relative to the bare LMG model. In particular, it leads to the appearance of a new stable phase-symmetric one, but with the total spin of the chain oriented in the reverse direction compared to the symmetric state of the bare LMG model. This phase might be stable as well as metastable, and the first order phase transition between two stable phases is possible. The broken symmetry phase might also become metastable at certain values of the parameters, which means that the corresponding phase transition becomes of first order as opposed to the second order in the case of the bare LMG model. Finally, coupling to the external spin might change the properties of the broken symmetry phase by splitting the corresponding minima of energy into two.

In case of an experimental attempt, it is possible to check the spin configuration of the coordinated Ising chain by using a well-developed and very sensitive method of polarized neutron reflectometry (PNR) [23–26] to improve the qubit quality, which is an important task [27–28].

The considered model is relevant in the field of quantum computation, as the layout is used for a certain realization of the CCZ gate [10]. Although, admittedly, the thermodynamic limit approximation made during the analysis is far from a feasible experimental setup involving only several qubits.

## Data Availability

All data that supports the findings of this study is available in the published article and/or the supporting information of this article.
